# SMOKING AND MODIC CHANGES IN PATIENTS WITH CHRONIC LOW BACK PAIN: A COMPARATIVE STUDY

**DOI:** 10.1590/1413-785220243205e278628

**Published:** 2024-10-28

**Authors:** Guilherme Augusto Foizer, Vagner Cleyton de Paiva, Carlos Gorios, Alberto Cliquet, João Batista de Miranda

**Affiliations:** 1. Universidade Estadual de Campinas, Campinas, SP, Brazil.; 2. Hospital Geral de Carapicuíba, Ambulatório de Coluna, Sao Paulo, SP, Brazil.; 3. Clínica Avançada de Ortopedia e Traumatologia, Sao Paulo, SP, Brazil.; 4. Centro Universitário São Camilo, Sao Paulo, SP, Brazil.; 5. Universidade Estadual de Campinas, Faculdade de Ciências Médicas, Departamento de Ortopedia, Reumatologia e Traumatologia, Campinas, SP, Brazil.

**Keywords:** Intervertebral Disc Degeneration, Intervertebral Disc, Tobacco Smoking, Low Back Pain, Magnetic Resonance Imaging, Degeneração do Disco Intervertebral, Dor Lombar, Disco Intervertebral, Tabagismo, Imagem por Ressonância Magnética

## Abstract

Objective: To compare the prevalence of smokers among patients with chronic low back pain, in the presence and absence of Modic changes, also the correlation between smoking history and progression of the Modic scale. Methods: Observational study, case-control type, with the inclusion of 340 vertebral segments in a total of 68 patients, separated into groups: with Modic (case group) and without Modic (control group). The odds ratio between the groups was verified using the Chi-Square test. Degree of correlation between smoking history (packs/year) and the degree of disc degeneration using Max-Modic and Sum-Modic, using Spearman’s non-parametric test. Results: The Modic group (MG) was 54% female and 46% male, with an average smoking history of 13.84 pack-years and an average of 1.42 altered segments per patient. Conclusion: An increased risk for Modic changes was found among smoking patients (odds ratio [OR] 4.09; 95% CI, 1.26-12.31; p < 0.01) and significant correlation between Max-Modic, sum-Modic and smoking history. ***Level of Evidence III, Retrospective comparative study.***

## RESUMO

Objetivo: Comparar a prevalência de tabagistas entre os pacientes com lombalgia crônica, na presença e na ausência de alterações de Modic, bem como a correlação entre carga tabágica e progressão da escala de Modic. Método: Estudo observacional, tipo caso-controle, com a inclusão de 340 segmentos vertebrais em um total de 68 pacientes, separados em grupos: com alterações de Modic (grupo caso) e sem alterações de Modic (grupo controle). A razão de chances entre os grupos foi verificada por meio do teste Qui-Quadrado. Grau de correlação entre a carga tabágica (maços/ano) e o grau de degeneração discal através do Máximo-Modic e a Soma-Modic, por meio de teste não paramétrico de Spearman. Resultados: O grupo Modic (GM) foi composto por 54% de pacientes do sexo feminino e 46% masculino, com média da carga tabágica de 13,84 anos/maço e média de 1,42 segmentos alterados por paciente. Conclusão: Foi encontrado um risco aumentado para alterações de Modic entre os pacientes tabagistas (razão de chances [OR] 4,09; IC 95%, 1,26-12,31; p < 0,01) e correlação significativa entre Máximo-Modic (r 0,3 p 0,01) e carga tabágica, assim como soma-Modic e carga tabágica (r 0,32 p 0,007). ***Nível de Evidência III, Estudo comparativo retrospectivo.***

## INTRODUCTION

 The clinical consequences of intervertebral disc degeneration have been highlighted as one of the main causes of pain and disability in the world ^1^ due to its potential role in chronic low back pain. ^2^ ^,^ ^3^

 Many theories suggest a causal relationship between smoking and chronic low back pain. Those theories include increased intra-abdominal pressure as a reflection of coughing, ^4^ changes in the perfusion of the intervertebral disc, ^5^ endocrine changes due to the effect of tobacco, ^6^ and changes in bone microtubular structures resulting from microfractures of the vertebral body. ^6^ ^,^ ^7^ . 

 Among the sequelae of disc degeneration, the signal intensity changes found in the vertebral endplates in magnetic resonance examination of the lumbar spine were proposed as a potential marker of chronic low back pain. The study published by Modic et coll. in 1988 found changes that were classified as Type I, with evidence of an increase in T2-weighted signal and a decrease in T1 signal, while Type II was characterized by an increased signal in T2 and T1. ^8^ ^,^ ^9^

Histologically in Type I lesions, the continuity of the endplate is disrupted, fibrous tissue replaces the bone marrow in this region amid thickened trabeculae, and the disc-bone interface is filled with vascularized granulation tissue. These changes represent edema and inflammation of the bone marrow. In addition to the above-mentioned findings of Modic Type I, samples of Modic type II also show replacement of bone marrow with adipose tissue. These findings represent the conversion of normal hematopoietic marrow into fatty, yellow bone marrow. Modic Type III is characterized by hypointense signs in both T1 and T2, related to subchondral bone sclerosis.

Although there is extensive research on the relationship between smoking and degenerative changes in the intervertebral disc, few studies specifically address the relationship between tobacco and Modic changes (MC), a fact that justifies research on the subject. The objective of this study was to compare the prevalence of smokers among patients with chronic low back pain, with and without the existence of Modic changes. As a secondary objective, we evaluated the existence of a correlation between higher smoking loads and a greater progression of the Modic scale.

## METHODOLOGY

### Study Type

This is an observational case-control study conducted at the Spine Orthopedics Outpatient Clinic at Carapicuíba General Hospital (HGC). We recruited patients in follow-up for chronic low back pain who received care sequentially between June 2018 and July 2019 and were not referred to surgical treatment. The study was approved by the Research Ethics Committee (CAAE 90700618.8.0000.0062). All research participants signed the Informed Consent Form (ICF – Appendix 1).

Inclusion criteria:

- Patients with low back pain for more than 12 weeks and;
- Having had an MRI examination of the lumbar spine with at least one degenerated ID.
- Exclusion criteria:
- Patients who did not wish to participate in the study;
- Previous brain and/or spinal surgeries;
- Spine disorders that lead to image changes on MRI examinations, such as vertebral fractures, spondylolisthesis, tumors or discitis.

### Clinical data

Firstly, socio-demographic data were collected by two orthopedists in personal, face-to-face interviews. In addition, the patients were asked if they had a smoking habit and, if they did, they were asked about their smoking loads. The smoking load was estimated as follows: “pack”/day x years. For example: 2 packs a day x 30 years = smoking load = 60 The body mass index was estimated by dividing mass by square height.

### Evaluation of magnetic resonance imaging

After the initial interview, patients were referred to another office where their MRI exams were evaluated by two orthopedists familiar with spine disorders. The orthopedists were blind to the initial interview. Each intervertebral segment of the lumbar spine was analyzed individually.

 T1 and T2-weighted MR images were analyzed in the sagittal plane and classified according to the study published by Modic et al. ^8^ For standardization purposes, when more than 5 lumbar vertebrae or transitional vertebrae characteristics were found, the last segment included in the study was L5-S1. 

### Statistical analysis

Initially, the data were analyzed by comparing the groups. The existence of Modic changes was considered the outcome. Patients who met the inclusion criteria and did not have exclusion criteria were separated into 2 groups: Modic (case group) and no Modic (control group). The odds ratio between the groups was evaluated using the Chi-Square test. Descriptive statistics were presented in absolute and relative frequencies, mean and standard deviation, median and interquartile, when appropriate.

The existence and the degree of correlation between smoking load (years/pack) and the degree of disc degeneration was also analyzed in two ways: maximum degree of Modic found in the vertebral segments (max-Modic) and the sum of the degrees of Modic of each individual (sum-Modic). Spearman’s nonparametric test was used.

 Correlations with adequate significance index (p < 0.05) were considered significant, and the Spearman coefficient was used to assess the strength of the correlation. Statistical analyses were performed in the *IBM SPSS Statistics v 23* software. 

## RESULTS

**Figure 1.**
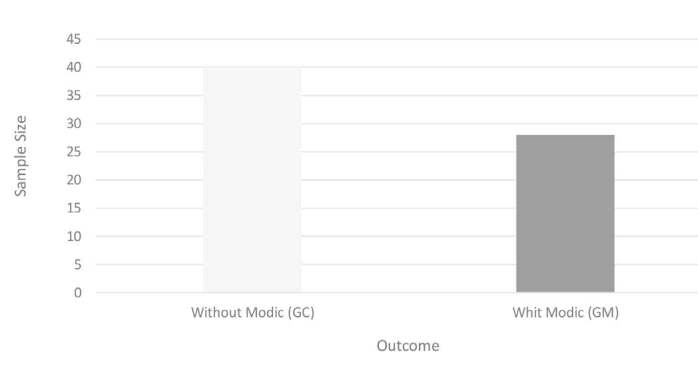
Sample size by outcome.

 A total of 340 vertebral segments in 68 patients were included in the study ( Figure 1 ). 

 The control group (CG) had 40 patients, with 30 female patients (75%) and 10 male patients (25%) ( table1 ). 

**Table 1.** Gender Vs Modic.

		MODIC		
GENDER		No	Yes	Total
F	Frequency	30	15	45
	Col pct	75.00	53.57	
M	Frequency	10	13	23
	Col pct	25.00	46.43	
Total		40	28	68

CHI-SQUARE TEST: X2=3.38; GL=1; P=0.066

The mean age of this group was 50.7 years with SD of 10.2 (19-69). The group had 33 non-smoking patients (82.5%) and 7 smokers (17.5%). The average smoking load in this group of 4.73 years/pack. The mean BMI was 27.46, SD 4.34 (18.9-37.3).

**Figure 2.**
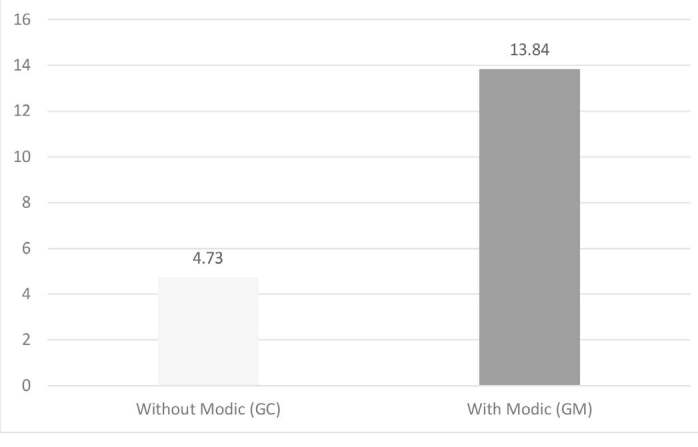
Average smoking load (packs/year).

 The Modic Group (MG) had 28 patients, with 15 female patients (54%) and 13 male patients (46%). The mean age was 50.57 years, with SD of 9.2 (32-69). In this group, 15 patients (54%) were non-smokers and 13 patients (46%) were smokers ( Table 2 ). The mean smoking load was 13.84 years/pack ( Figure 2 ). The mean BMI was 27.3, SD of 3.94 (20.6-37.6). 

**Table 2.** Smokers Vs Modic.

Smoker No		MODIC		Total
		Yes		
No	Frequency	33 **(82.5%)**	15 (53.5%)	48
Yes	Frequency	7 (17.5%)	13 ( **46.4%)**	20
Total		40	28	68

CHI-SQUARE TEST: X2 = 6.64; GL = 1; **P = 0.010**

**OR=4.09; 95% CI OR: (1.36; 12.31)**

**Figure 3.**
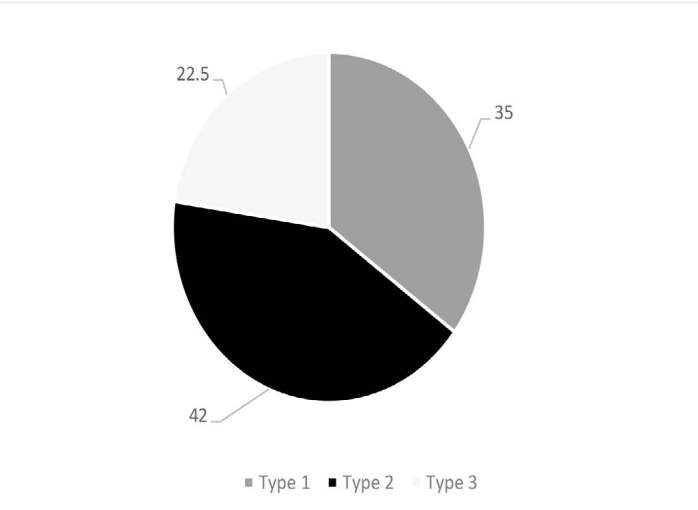
Frequency of Modic change type in sample.

 Among these 28 patients, 40 vertebral segments with MC were found (mean of 1.42 changed segments per patient) ( Table 3 ). The most affected was L5S1, accounting for 42.5% (17 cases) of the total MC, followed respectively by L4L5 with 30% of segments (12 cases), L3L4 and L2L3 with 12.5% of segments (5 cases), and L1L2 with 2.5% (1 case) ( figure3). 

**Figure 4.**
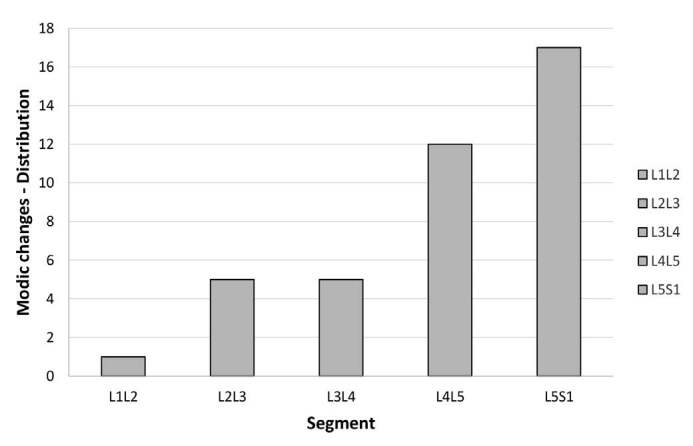
Modic changes (distribution per segment).

 Considering the types of changes, Modic Type 2 was the most frequent with 42% of the total MC, followed by Type 1 with 35% (14 cases), and Type 3 with 22.5% (9 cases) ( Figure 4 ). 

An increased risk for Modic changes was found among smoking patients (odds ratio [OR] 4.09; 95% CI, 1.26-12.31; p < 0.01).

**Figure 5.**
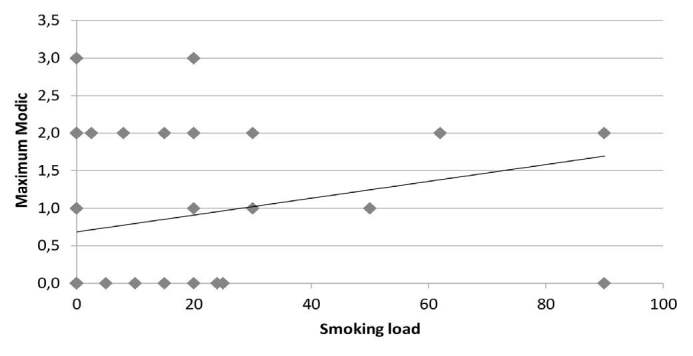
Correlation between Smoking Load and Maximum Modic.

**Figure 6.**
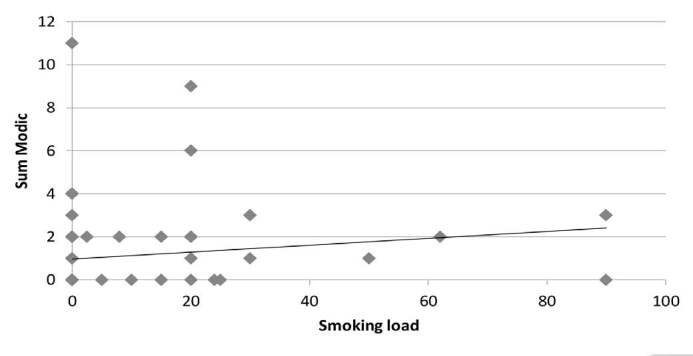
Sum Modic Vs Smoking Load.

 The correlation tests between smoking load and max-Modic per patient showed a Spearman’s r of 0.3 with a significance index of 0.01 ( Figure 5 ), while the correlation tests between smoking load and sum-Modic showed a Spearman’s r of 0.32 with a significance index of 0.007 ( Figure 6 ) 

**Table 3.** Modic distribution (frequency and percentage).

**Modic-Frequency**					**Modic-Percentage**				
	**0**	**1**	**2**	**3**		**0**	**1**	**2**	**3**
L1-L2	67	1			L1-L2	98.53	1.47		
L2-L3	63	3	1	1	L2-L3	92.65	4.41	1.47	1.47
L3-L4	63	2	1	2	L3-L4	92.65	2.94	1.47	2.94
L4-L5	56	4	5	3	L4-L5	82.35	5.88	7.35	4.41
L5-S1	51	4	10	3	L5-S1	75.00	5.88	14.71	4.41
Max Modic	40	8	15	5	Max Modic	58.82	11.76	22.06	7.35

## DISCUSSION

 There has been a long discussion about the potential role of smoking in the etiology of chronic low back pain. A study in Finland compared disc degeneration rates between 20 pairs of monozygotic twins, with and without a history of smoking ^10^ . The study found 18% more disc degeneration among smokers and an important difference between the degree of obstruction by arteriosclerosis in carotid ultrasound. 

 Therefore, it is believed that that situation may lead to lower tissue blood flow and subsequent lower repair power of the tissue around the intervertebral disc. ^11^

 The results showed a degree of homogeneity between the groups regarding demographic factors. This ruled out confounding factors that are frequent in studies on the etiology of degeneration of vertebral elements. Prevalence between sexes, for example, is a controversial topic usually based on exposure factors more commonly associated to each sex, such as heavy work, obesity and smoking. ^7,12—14^ Similarly, the mean age of the groups (both 50.7 years old) ruled out a possible confounding bias in the comparison, considering the association between age and MC to be common. ^7^

The percentage of smokers in the Modic group was significantly higher, as was the smoking load, compared to the control group. The smoking load of the studied group was almost three times higher than that of the control group. This datum was the most discrepant between the groups, given that the other commonly studied association factors did not differ statistically from each other in this study.

 Studies involving patients with Modic changes are usually cross-sectional and non-comparative. Among them, Arana et al. ^13^ found MC in 81% of their sample, composed of clinical patients with 50% smokers, while Mera et al. showed 65% prevalence, but did not evaluate smoking. ^12^ Although our prevalence of MC was lower in the total sample (41%), we found 46% prevalence of smokers in the outcome group. 

 Regarding the distribution of MC by segments, increased prevalence was found at more caudal levels (72.5% of cases were located between L4L5 and L5S1). This suggests that the load and mobility to which the vertebral segments are subjected have an effect on the formation of MC. This finding was similar to studies published in the field, in which a higher prevalence of MC is found in more caudal segments. ^7^ ^,^ ^13^ ^,^ ^15^ ^,^ ^16^ . 

 Among the types of changes, Modic type 2 was the most frequent. This change was described as an intermediate process with a degree of chronicity. ^9^ Many authors ^13^ ^,^ ^14^ ^,^ ^17^ have described a higher prevalence of Modic type 2, possibly related to the progressive nature of the degeneration process. It is also known that samples containing patients with low back pain for a shorter time or higher degrees of pain have increased levels of Modic type 1 due to its greater inflammatory nature. ^12^ . 

 Other authors had already evaluated the odds ratio of the association between MC and smoking. Leboeuf-Yde et al. showed a relationship between MC involving heavy work in combination with heavy smoking ^14^ . The odds ratio for MC in smokers was 4.9, similar to that found in our study, but this analysis was performed by dividing smoking into heavy, light and non-smoking groups. Our study used the smoking load in years/pack, as did Arana et al. ^13^ . However, unlike all others, we correlated the variables using Spearman’s test. Thus, the most important datum in this study was the correlation between higher smoking loads and higher degrees of Modic. This is relevant because it shows that, in addition to the association between the variables, a correlation can be further investigated, thus showing that smoking can have an effect not only on the formation of MC, but also on their evolution to endplates turning fatty and sclerotic. This analysis allows for more objectivity in the interpretation of results and in the replication of the methods in new studies. 

 As limitations, we can mention the retrospective nature of this study, which, along with the absence of sample calculation, does not allow a detailed analysis of the several risk factors that may be involved in the etiology of MC. Although there is good inter and intraobserver agreement for the Modic classification described in the literature, ^18^ it was not performed in this study. Nevertheless, the results clearly show that smoking has a more important role in patients with MC, demonstrating that complementary research may be welcome. 

## CONCLUSION

In the studied sample, there was a higher chance for the existence of smokers among patients with Modic changes. A correlation was found between higher smoking loads and higher degrees of Modic.
